# Securinine, a Myeloid Differentiation Agent with Therapeutic Potential for AML

**DOI:** 10.1371/journal.pone.0021203

**Published:** 2011-06-24

**Authors:** Kalpana Gupta, Amitabha Chakrabarti, Sonia Rana, Ritu Ramdeo, Bryan L. Roth, Munna L. Agarwal, William Tse, Mukesh K. Agarwal, David N. Wald

**Affiliations:** 1 Department of Pathology, Case Western Reserve School of Medicine, Cleveland, Ohio, United States of America; 2 Invenio Therapeutics, Cleveland, Ohio, United States of America; 3 Departments of Pharmacology and Medicinal Chemistry, University of North Carolina Chapel Hill Medical School, Chapel Hill, North Carolina, United States of America; 4 Department of Genetics, Case Western Reserve School of Medicine, Cleveland, Ohio, United States of America; 5 Department of Medicine, West Virginia University, Morgantown, West Virginia, United States of America; 6 University Hospitals Case Medical Center, Cleveland, Ohio, United States of America; University of Texas Southwestern Medical Center at Dallas, United States of America

## Abstract

As the defining feature of Acute Myeloid Leukemia (AML) is a maturation arrest, a highly desirable therapeutic strategy is to induce leukemic cell maturation. This therapeutic strategy has the potential of avoiding the significant side effects that occur with the traditional AML therapeutics. We identified a natural compound securinine, as a leukemia differentiation-inducing agent. Securinine is a plant-derived alkaloid that has previously been used clinically as a therapeutic for primarily neurological related diseases. Securinine induces monocytic differentiation of a wide range of myeloid leukemia cell lines as well as primary leukemic patient samples. Securinine's clinical potential for AML can be seen from its ability to induce significant growth arrest in cell lines and patient samples as well as its activity in significantly impairing the growth of AML tumors in nude mice. In addition, securinine can synergize with currently employed agents such as ATRA and decitabine to induce differentiation. This study has revealed securinine induces differentiation through the activation of DNA damage signaling. Securinine is a promising new monocytic differentiation inducing agent for AML that has seen previous clinical use for non-related disorders.

## Introduction

Acute myeloid leukemia (AML) is the most common type of acute leukemia with over 13,000 cases expected in the United States this year, mostly in older adults [1], [2]. The current therapeutic regimen for AML involves traditional chemotherapeutics which unfortunately have poor efficacy and high toxicity. This is especially problematic in elderly patients as they are not able to tolerate the existing chemotherapeutic agents and they are left with no satisfactory therapeutic options. Currently the 5 year survival in the over 65 age group is only 4% while it is 33% for those under 65 [2], [3].

AML is a broad range of disorders that all have the defining feature of leukemic cells with a maturation arrest. The poor efficacy of the current AML therapeutics is likely due to the pathophysiology of AML as it is characterized by both the arrest of differentiation of immature myeloid cells as well as rapidly dividing cells. Unfortunately, the current AML chemotherapeutics such as cytarabine and idarubicin primarily target the highly proliferative cells. By instead inducing terminal differentiation, leukemic cells can be forced to lose their ability to proliferate and eventually die off without the necessity for overt cytotoxicty.

The clinical potential of differentiation therapy for AML has been demonstrated for one rare subtype of AML, acute promyelocytic leukemia (APL) (5–10% of AML). In this case the differentiation-inducing agent all-trans retinoic acid (ATRA) has revolutionized the treatment of APL and leads to the long term survival and presumed cure of 75–85% of patients [4]. In addition as ATRA is more tolerable for elderly patients, this group can still achieve good outcomes with regimens consisting of ATRA and low dose chemotherapy. Unfortunately ATRA is not clinically useful for other subtypes of AML. Through a compound library screen performed to identify new AML differentiation-inducing compounds, we discovered a plant derived compound, securinine, which exhibits potent leukemia differentiation-inducing activity.

Securinine is the major alkaloid natural product from the root of the plant *securinega suffruticosa*. It was originally discovered over 50 years ago and reported to have potent biological activity [5]. Though this compound has not been utilized in the United States, it has been used clinically in several other countries particularly China and Russia. In China it is considered one of the 50 fundamental Chinese herbs and is used in Chinese herbal medicine [6]. Securinine has been found to be active as a γ-amino butyric acid (GABA) receptor antagonist [7]. Its activity as a GABA antagonist, likely explains its reported clinical success in limited studies for the treatment of neurological conditions such as amyotrophic lateral sclerosis (ALS), poliomyelitis and multiple sclerosis [8], [9], [10]. Though securinine has never been previously tested clinically as an anti-cancer agent, there is a single report that demonstrates that it can induce apoptosis at high doses in a leukemic cell line [11]. In addition, we have recently found that securinine may have potential utility against certain types of colon cancer [12]. Finally, securinine has also been reported to induce macrophage activation and therefore proposed as a potential therapeutic for infectious processes [13]. Here we demonstrate that securinine can induce AML differentiation and has potential as an AML therapeutic.

## Results

### Discovery of Securinine

**Figure 1:**
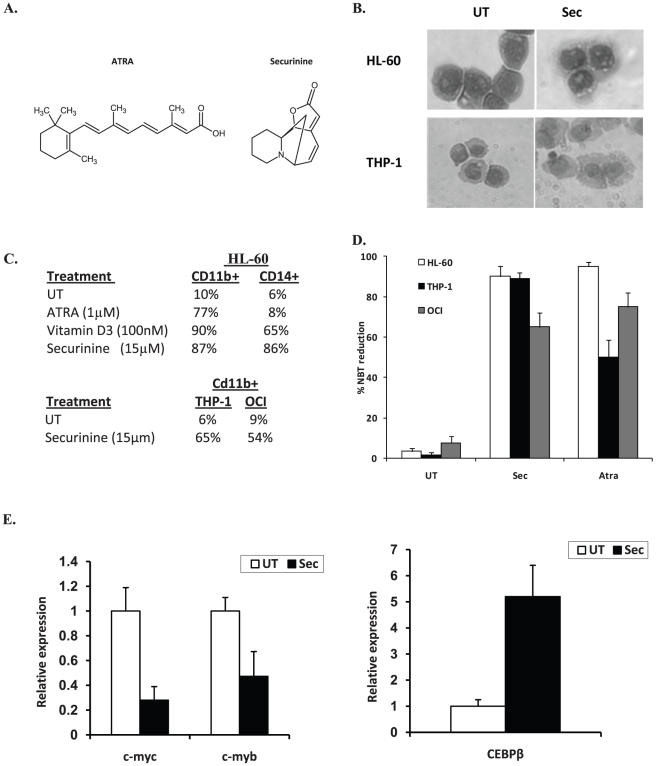
Securinine induces monocytic differentiation of AML cells. A. Chemical structure of Securinine and ATRA. B. Securinine induces morphologic changes consistent with monocytic differentiation. After treatment for 4 days with securinine (15 µM), cytospin preparations were prepared and HL-60 and THP-1 cells were stained with Wright-Giemsa stain. C. Securinine induces immunophenotypic changes consistent with monocytic differentiation. After treatment for 4 days with securinine (15 µM), HL-60 cells were stained with CD11b-PE and CD14-FITC, THP-1 and OCI-AML3 cells were stained with CD11b-PE and flow cytometric analysis was performed. D. Securinine induces potent NBT reduction activity consistent with myelomonocytic differentiation. HL-60, OCI-AML3 and THP-1 cells were treated with securinine (15 µM) or ATRA (1 µM) for 4 days and then the NBT reduction assay was performed by stimulating cells with 200 ng/ml of PMA in the presence of 5 mg/ml NBT for 20 minutes. The percentage of NBT positive cells (blue cells) was calculated by counting at least 200 cells under a light microscope. E. Securinine regulates the expression of transcription factors with known roles in AML differentiation. HL-60 cells were treated with securinine (15 µM) or vehicle for 24 hours and the relative expression of the indicated genes was measured by real-time PCR.

In order to identify clinically useful differentiation-inducing agents, a small molecule compound library screen was performed using a collection consisting predominantly of drugs that have previously been used in humans (Prestwick and Lopac collection). The phenotypic screen assessed differentiation by measuring the functional maturation of HL-60 leukemic cells by the nitroblue tetrazolium (NBT) reduction assay, a test that is highly specific to and has been used extensively as a measure of functional myelomonocytic differentiation [14], [15], [16], [17], [18]. The NBT reduction test measures the ability of cells to generate a respiratory burst, a function that is only present in differentiated cells. As only live cells reduce NBT, this screen is heavily biased in identifying potent, yet non-toxic agents. Specific details regarding the screen and the “hits” resulting from this screen have been described previously [19]. From this screen securinine was identified as the most potent inducer of differentiation. Interestingly, securinine was also identified as a strong “hit” during later screening efforts utilizing the NCI diversity set. Securinine, an alkaloid with a unique ring structure, is structurally unrelated to any previously reported myeloid differentiation inducers, but interestingly it has seen clinical use for non-cancer related conditions (figure 1a).

### Securinine induces AML differentiation

Securinine exhibits clinical potential as an AML differentiation agent partially due to its ability to differentiate a wide variety of AML cell lines as well as patient samples. To confirm securinine induces AML differentiation, three AML cell lines were investigated, HL-60 cells that typically undergo granulocytic or monocytic differentiation and THP-1 and OCI-AML3 cells that undergo monocytic differentiation. These cell lines were examined by morphology, immunophenotyping, and NBT reduction (figure 1b–d). Morphologically, securinine induces monocytic differentiation as can be seen from an increased cytoplasmic to nuclear ratio. In addition, HL-60 cells exhibit a condensed and indented nucleus while THP-1 cells become significantly larger in size (figure 1b). No significant morphologic changes were observed with OCI-AML3 cells, likely as they already have a predominant monocytic morphology.

Immunophenotyping was performed to confirm monocytic differentiation in HL-60 cells as this cell line, unlike THP-1 and OCI-AML3 cells, differentiates along several different pathways. This analysis confirmed monocytic differentiation as securinine treatment leads to the upregulation of the cell surface markers CD11b, which is upregulated during myelomonocytic differentiation, as well as CD14 which is upregulated primarily during monocytic differentiation. In comparison, ATRA which induces granulocytic differentiation induces primarily CD11b expression while vitamin D3 which induces monocytic differentiation upregulates both CD11b and CD14 (figure 1c). Similarly, in THP-1 and OCI-AML3 cells which only undergo monocytic differentiation, maturation was confirmed by CD11b expression that increased from 11% to 73% and 15% to 64% respectively after 96 hours of securinine treatment (15 µM). Besides, morphology and immunophenotyping, securinine exhibits high differentiation activity in HL-60, OCI-AML3, and THP-1 cells as measured by NBT reduction. For example, securinine leads to NBT reduction in 90%+/−5 of HL-60 cells and 89%+/−3 of THP-1 cells (figure 1d).

### Confirmation of Securinine-mediated differentiation

In order to begin to elucidate pathways through which securinine induces AML differentiation, a gene expression microarray experiment was performed to identify gene expression changes in HL-60 cells after securinine treatment. As the differentiation process itself leads to changes in the expression of thousands of genes which help define the phenotype of the differentiated cells, but fail to give significant insights into securinine's mechanisms of action, our initial analysis of this data was focused on genes with a known function in transcription. This analysis identified 158 genes whose expression level was modulated at least 2.5 fold in response to securinine treatment (24 hours) as compared to vehicle treated cells (see Figure S2). Many of these genes include transcription factors that are well known to modulate AML differentiation such as c-myc and c-myb which are downregulated and CEBP/β, CEBP/δ, egr-1, mafB, fos, and jun that are upregulated. These findings further support securinine induces myeloid differentiation.

In order to validate the gene array results, the upregulation of the myeloid transcription factor, CEBP/β, and downregulation of proliferation related gene c-myc and transcription factor c-myb were analyzed by real-time PCR. As can be seen in figure 1e, the securinine-mediated upregulation of CEBP/β and downregulation of c-myc and c-myb were confirmed.

### Securinine induces terminal differentiation

**Figure 2:**
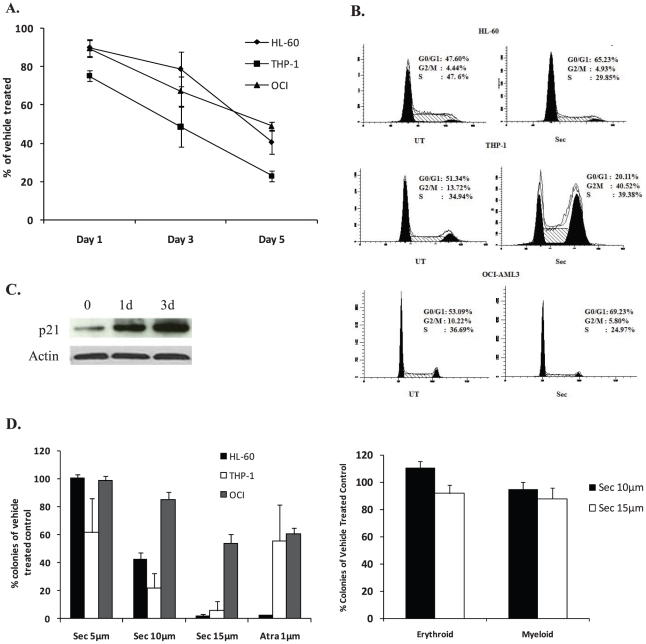
Securinine leads to alterations in AML cell proliferation. A. Securinine inhibits AML cell proliferation. HL-60, OCI-AML3 and THP-1 cells were treated with vehicle or securinine (15 µM) for 5 days. The number of cells present at days 1, 3, 5 after treatment was determined by counting at least 200 cells with a hematocytometer. Results are expressed as percentage of cells present in the treated as compared to vehicle wells. Results are an average of 3 independent experiments. B. Securinine induces alterations in the cell cycle of HL-60, OCI-AML3 and THP-1 cells. AML cells were treated for 24 hours with the securinine (15 µM) or vehicle, fixed, stained with propidium iodide and analyzed for cell cycle content by flow cytometry. Results are representative of 3 independent experiments. C. Securinine induces p21. HL-60 cells were treated with securinine (15 µM) for the indicated timepoints and cell lysates were analyzed for p21 expression by western blot. D. Securinine potently inhibits colony formation of AML, but not normal hematopoietic cells. HL-60, OCI-AML3 and THP-1 cells were incubated with securinine or vehicle for 4 days and the drug was washed off. An equal number of viable cells were added to soft agar and colony formation was assessed after 10 days. Normal human bone marrow was treated in a similar manner, but was plated in methylcellulose. Results are expressed as the percentage of colonies in the treated group compared to vehicle control group and are an average of two independent experiments performed in duplicate.

To gain a better understanding of securinine's activity on leukemic cells, its effects on leukemic cell growth of HL-60, THP-1, and OCI-AML3 cells were investigated. Securinine led to a significant inhibition of cell growth in all cell lines as compared to the vehicle control (figure 2a). For example, in THP-1 cells at 5 days after treatment securinine led to only 22%+/−2.6 of the proliferation seen in vehicle treated cells (figure 2a). In order to further understand how securinine induces growth inhibition, cell cycle analysis was performed. Though securinine leads to significant alterations in the cell cycle, the specific alterations are cell type specific. Securinine growth inhibition of HL-60 cells involves an accumulation of cells in the G0/G1 phase of the cell cycle with 65% of cells present in G0/G1 as compared to 47% in the vehicle control at 24 hours after treatment (figure 2b). Consistent with accumulation in the G0/G1 phase, securinine induces p21 that is known to play an important role in preventing the G1 to S transition (figure 2c). In contrast to HL-60 and OCI-AML3 cells, in THP-1 cells, securinine leads predominantly to accumulation of cells in the G2/M phase with 41% in the securinine treated cells as compared to 14% present in vehicle treated cells after 24 hours of treatment.

### Securinine induces dramatic growth inhibition

Though securinine induces evidence of differentiation, for differentiation agents to be clinically useful they must lead to significant growth arrest. Further this growth inhibition must result in viable cells maintaining a loss of proliferative capacity even when drug is washed off. To assess whether the observed securinine-mediated growth inhibition and differentiation results in terminal differentiation, colony forming assays were performed in soft agar. Limited exposure (96 hours) of THP-1 and HL-60 (and to a lesser extent OCI-AML3 cells) to securinine at doses greater than 10 µM led to dramatic reductions in colony formation in soft agar demonstrating that terminal differentiation is induced (figure 2d). In contrast to the significant inhibition of colony formation in AML cells, treatment with securinine did not lead to a significant change in the growth of colonies from normal human bone marrow (figure 2d). In addition, it did not appear to affect the differentiation of normal human bone marrow progenitor cells along the myeloid or erythroid lineages. As expected, securinine did not lead to any significant difference in cell death at the doses utilized as compared to vehicle treated cells.

### Securinine induces differentiation in a wide range of AML cell lines and primary patient samples

**Figure 3:**
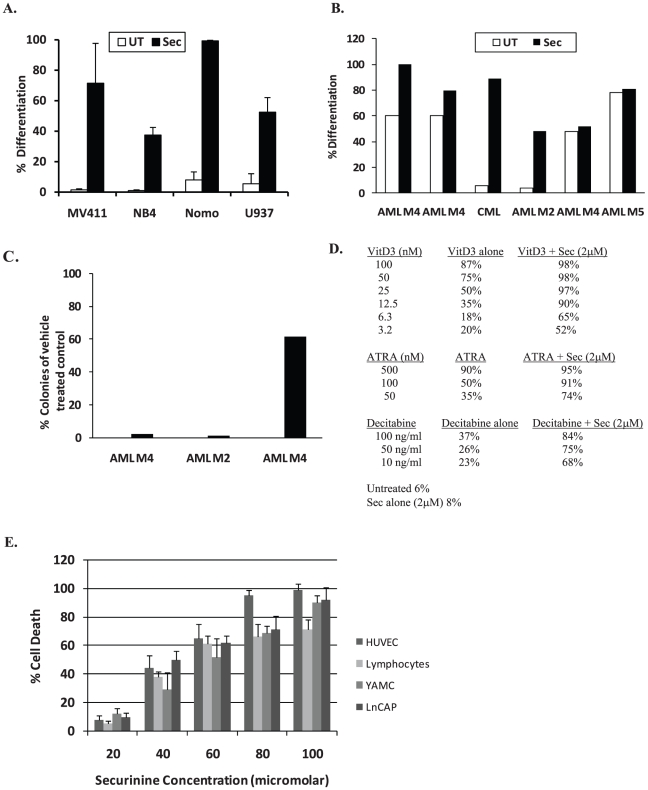
Securinine has differentiation activity in multiple AML cell lines and patient samples and can synergize with clinically used therapeutics. A. Securinine induces differentiation in multiple leukemic cell lines. The indicated cell lines were treated with vehicle, securinine (15 µM) or ATRA (1 µM) for 4 days and the NBT reduction assay was performed. Results are representative of 3 independent experiments. B. Securinine can induce differentiation in primary patient samples. Primary leukemic blasts derived from the peripheral blood of patients with active leukemia disease were purified by flow sorting after staining with CD34-PE if the leukemic blast percentage was less than 70%. Cells were treated with securinine (15 µM) or vehicle for up to 6 days and assessed for differentiation by NBT reduction. AML subtype is indicated in the figure. C. Securinine can induce significant growth inhibition in primary patient samples. Primary leukemic blasts were treated for 5 days with securinine, the drug was washed off and the cells were plated in methylcelluose and assessed for colony formation after 10 days. AML subtype is indicated in the figure. D. Securinine can synergize in HL-60 cells with Vitamin D3, ATRA, and Decitabine. To assess Vitamin D3, ATRA and decitabine synergy, HL-60 cells were treated with the indicated compounds for 4 days and the NBT reduction assay was performed. Results are representative of 3 independent experiments. E. Securinine exhibits low *in vitro* toxicity. The indicated cell lines were treated with up to 100 µm of securinine for 3 days and cell death was assessed. Results represent three independent experiments.

Besides HL-60, OCI-AML3, and THP-1 cells, one of the most promising properties of securinine is its ability to differentiate a wide variety of AML cell lines and patient samples. For example, securinine induces evidence of differentiation in NB4, U937, NOMO-1, and MV-411 cells (figure 3a). In addition securinine can also induce evidence of differentiation in primary patient leukemic samples (figure 3b). Securinine induced differentiation in 4 out of 6 leukemia patient samples tested as measured by NBT reduction. In one of the two patient samples that did not exhibit securinine-mediated differentiation, the cells instead underwent securinine-mediated cell death. Interestingly, securinine was able to induce differentiation in cells from a patient with chronic myeloid leukemia indicating that it may also be useful for this closely related myeloid disorder. Several AML samples were also tested for the induction of growth inhibition by colony assays and securinine was found to significantly reduce the ability of primary AML cells to form colonies in 3 out of the 4 patient samples tested (figure 3c).

### Securinine synergizes with other agents with potential clinical use for AML

Though ATRA is a successful differentiation agent for a subset of AML patients, it is only clinically efficacious when combined with low dose chemotherapy. Therefore, we investigated combination therapies of securinine with other clinically used agents. Securinine can synergize with several other differentiation-inducing agents including ATRA, vitamin D, and decitabine suggesting that these agents may work through different pathways and raising the possibility that securinine may be useful as part of a combination therapy (figure 3d). Co-treatment of HL-60 cells with a low dose of securinine (2 µM) led to an increase in NBT reduction from 6–8% with securinine alone to 91% with ATRA (100 nM), 84% with decitabine (100 ng/ml) and 98% with 1,25 dihydroxyvitamin D3 (50 nM). Similar results were obtained using other AML cell lines (data not shown). This work raises the possibility that securinine may enhance the clinical activity of ATRA and decitabine as well as potentially allow the use of levels of Vitamin D3 that do not cause toxic hypercalcemia.

### Securinine exhibits low in vitro toxicity

In order for securinine to be clinically useful for AML, it must induce preferential effects on AML cells. Utilizing a panel of normal and cancer cells, AML differentiation occurs at several fold lower doses (10–15 µM) than the LD_50_ doses at three days after treatment in the other cell types tested (45–60 µM) (figure 3e). In addition, as mentioned previously, securinine did not significantly impair the growth of colonies from normal bone marrow.

### Securinine demonstrates in vivo activity

**Figure 4:**
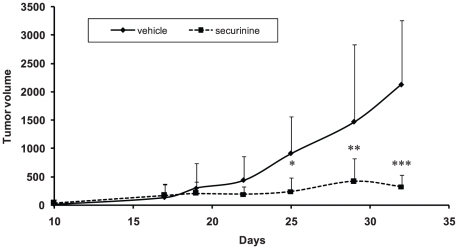
Securinine significantly inhibits the growth of established AML tumors in nude mice. Results shown represent the average volume of tumors from the mice measured during the study period. * p = 0.0075; ** p = 0.046; *** p<0.001.

In order to assess securinine's potential as an AML therapeutic, mouse xenograft experiments were performed. For these experiments, the effect of securinine on inhibiting the growth of established HL-60 cell subcutaneous tumors in nude mice was assessed. In this tumor model, tumor growth was significantly impaired with securinine treatment indicating it has potential as an AML therapeutic (figure 4). Securinine treated mice (n = 5 mice, bilateral tumors), exhibited an average of more than 75% smaller tumors than vehicle treated mice at the end of the study period. Due to the extremely short half life of securinine (estimated to be significantly less than 30 minutes), these experiments utilized frequent securinine administration (3 times a day) to show efficacy. A second study performed using 2 times a day dosing showed similar results (70% smaller tumors at the end of the study period). Initial experiments performed with once a day administration did not show a statistically significant effect on tumor growth. Though we utilized frequent dosing of securinine, we did not observe any evidence of mouse toxicity (including seizures) or weight loss.

### Securinine-mediated differentiation does not involve GABA receptor inhibition

As securinine's clinical use for neurological diseases is based upon its well known function as a GABA receptor antagonist and leukemic cells (including HL-60 cells) are known to express this receptor, we assessed whether or not this activity plays a role in securinine-mediated differentiation [20]. Other GABA antagonists such as picrotoxin, bicuculline, and flumazenil do not exhibit differentiation-inducing activity in HL-60 cells suggesting that securinine is likely working through a different mechanism (data not shown). In addition, virosecurinine and allosecurinine which are stereoisomers of securinine that do not exhibit as high an affinity for the GABA receptor as securinine were found to induce relatively similar differentiation of HL-60 cells [7], [21], [22]. For example, allosecurinine (15 µM) and virosecurinine (15 µM) induced differentiation in HL-60 cells with allosecurinine differentiating 80% and virosecurinine 95% of cells as measured by the NBT reduction test. As securinine's interaction with the GABA receptor is responsible for its main clinical side effect, seizures, it is quite likely that this molecule can be modified to maintain AML differentiation activity while minimizing its seizure effects.

### Securinine induces DNA damage signaling

**Figure 5:**
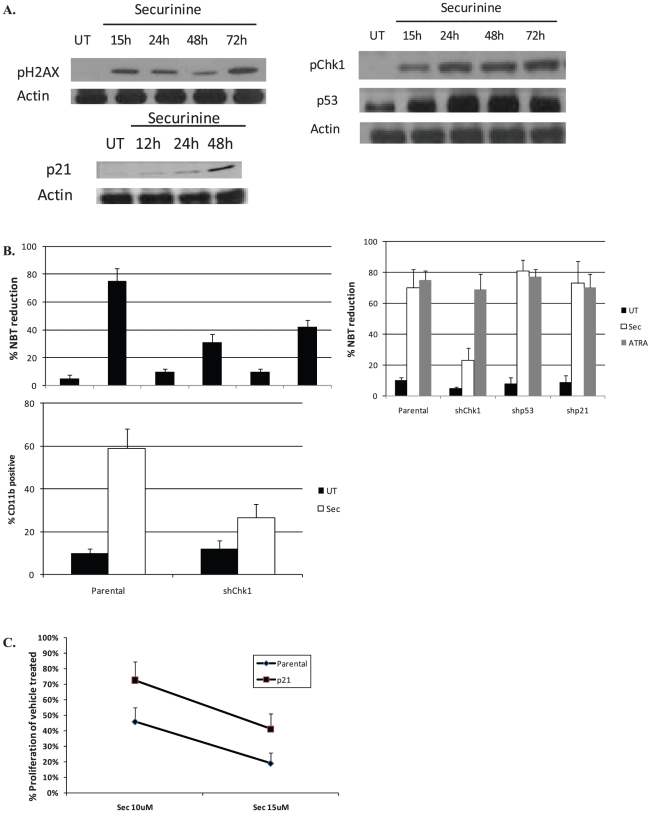
Mechanisms of securnine-mediated differentiation. A. Securinine activates DNA damage signaling. OCI-AML3 cells were treated with securinine (15 µM) for the indicated times and cell lysate was prepared. The lyaste was analyzed for the proteins shown by western blot. B. Inhibition of DNA damage signaling significantly impairs securinine-mediated differentiation. First panel: OCI-AML3 cells were treated with UCN-01 (1 µM), caffeine (1 mM), securinine (15 µM), or a combination for 4 days and the NBT reduction assay was performed. Results are an average of 3 experiments. Second Panel: OCI-AML3 cells stably infected with shp53, shChk1, shp21, or a vector control were treated with securinine (15 µM) for 4 days and the NBT reduction assay and CD11b expression was assessed. C. p21 impairs securinine-mediated growth arrest but not securinine-mediated differentiation. OCI-AML3 cells stably infected with shp21 or vector control were treated with the indicated doses of securinine and were counted after 72 hr using a Coulter Counter.

In order to further assess how securinine leads to differentiation, we investigated its effects on DNA damage signaling as we previously observed securinine induces weak DNA damage in colon cancer cells [23]. Interestingly, even though non-cytotoxic doses of securinine were utilized, securinine induced limited DNA damage and the subsequent activation of DNA damage signaling in leukemic cells (figure 5). While the activation of DNA damage signaling is well known to lead to growth arrest as a protective response in many cell types, many pathways involved in this response overlap with AML differentiation mechanisms. For example, p53 and p21 have both been associated with growth arrest and AML differentiation [24], [25]. After treatment of AML cells with securinine, we observed limited DNA damage (as measured by H2AX phosphorylation) and the activation of DNA damage signaling (as measured by Chk1 phosphorylation and the induction of p53 and p21) (figure 5a).

### Inhibition of DNA damage signaling impairs securinine-mediated differentiation

Upon disruption of DNA damage signaling with chemical inhibitors or the knockdown of Chk1 using shRNA, securinine-mediated (but not ATRA-mediated) differentiation is greatly attenuated (figure 5b). For example, pre-treatment of OCI-AML3 cells with caffeine (ATM/ATR inhibitor) or with UNC-01 (Chk1/2 inhibitor) significantly impaired securinine-mediated differentiation. In addition the knockdown of Chk1, led to a significant decrease in securinine mediated, but not ATRA mediated-differentiation as measured by CD11b expression and NBT reduction (figure 5b). In contrast, despite the nearly complete knockdown of p53 expression in OCI-AML3 cells, there was no impairment in securinine-mediated differentiation. Of note HL-60 cells, which are p53-null, are highly susceptible to securinine-mediated differentiation suggesting that securinine differentiation is independent of p53.

### p21 is important for securnine-mediated growth inhibition, but not differentiation

As p21 is known to play an important role in both growth inhibition and differentiation, the role of p21 in mediating securinine's biological effects was analyzed. In order to assess the importance of p21 in securinine's activities, p21 was knocked down using lentiviral shRNA in OCI-AML3 cells. Surprisingly, as can be seen from figure 5c, knockdown of p21 had no effect on securinine-mediated differentiation. In contrast, p21 deficiency impairs securinine-mediated growth inhibition suggesting it plays a role in securinine-mediated growth inhibition, but not differentiation.

## Discussion

We have identified a promising new AML differentiation agent, securinine, through a compound library screen. Securinine is a plant-derived alkaloid that has already seen clinical use for primarily neurological conditions. Due to its prior clinical use and its ability to differentiate a wide range of AML cell lines as well as patient samples, securinine is a promising new therapeutic candidate for AML.

New therapeutics for AML that are efficacious for even a small subset of patients are highly desirable due to the poor efficacy and high toxicity of the existing AML chemotherapeutics that have not undergone significant change over the last 30 years. The potential of AML differentiation agents has been demonstrated through the use of ATRA that has seen remarkable clinical success for a small number of AML patients that have the Acute Promyelocytic Leukemia (APL) subtype. Unfortunately ATRA is not efficacious for the majority of AML patients that fall into the other AML subtypes. Securinine's ability to enhance ATRA-mediated differentiation particularly in non-APL cell lines leads to the possibility that it may allow ATRA to be used for a wider subset of patients. In addition securinine can synergize with other agents such as decitabine that alone induces weak differentiation as well as vitamin D which though a potent differentiation compound leads to toxic hypercalcemia at the doses required for its activity as a single agent. Securinine's ability to synergize with several agents likely relates the fact that it induces differentiation through a different mechanism.

Our studies suggest that the mechanism of securinine-mediated differentiation involves limited DNA damage that leads to the activation of DNA damage signaling. As DNA damage signaling is intended as a protective response, it can lead to growth arrest or cell death dependent upon the amount of damage and the specific cell type. As leukemia cells have a propensity to differentiate, these cells can undergo differentiation after limited DNA damage in addition to growth arrest. Securinine-mediated differentiation was found to be dependent upon an ATM/ATR and Chk1, but independent of p53 and p21. Interestingly, we found that p21 was important for securinine-mediated growth inhibition, but not differentiation suggesting a bifurcation of the AML differentiation response in the DNA damage signaling pathway upstream of p21.

Securinine exhibits *in vivo* activity as demonstrated through an AML mouse xenograft model system with no evidence of toxicity at the doses utilized. In previous clinical studies investigating securinine's potential for neurological diseases, the primary side effect reported was seizures due its known activity as a GABA_A_ receptor inhibitor which is thought to be important for its efficacy. Interestingly, securinine's GABA activity does not appear to be involved in its AML differentiation activity suggesting that securinine can be further modified as an AML therapeutic to minimize its potential for causing seizures. Work is currently in progress to identify securinine analogues that have this desired activity. Overall, these studies demonstrate that securinine's potential as an AML therapeutic and identify novel mechanisms of action of this compound that lead to its activity as a differentiation agent.

## Materials and Methods

### Ethics

The Case Western Reserve University Animal Research Committee approved all of the animal protocols used in this study.

### Chemicals

1,25 dihydroxyvitamin D3, ATRA, Nitroblue tetrazolium (NBT), UCN01 and PMA were purchased from Sigma. Securinine was purchased from LKT Labs. Bisindolylmaleimide (BIS) was from Calbiochem.

### Cell Lines

All cell lines were obtained from ATCC except OCI-AML3 cells were from DSMZ and YAMC cells were provided by Dr. Sandy Markowitz. Patient samples were obtained from the stem cell core facility at University Hospitals Case Medical Center.

Cells were cultured in IMDM or RPMI-1640 media (Invitrogen) except for HCT116, 293T (DMEM) and HUVEC (EGM Lonza). Media was supplemented with 10% FBS (Invitrogen), penicillin G (100 µg/ml) and streptomycin (100 µg/ml). Mononuclear cells were separated by Ficoll-Hypaque (Sigma) density-gradient centrifugation and for selected patients leukemic myeloblasts were isolated by flow sorting after staining with anti-CD34 PE (Beckton Dickinson). The work on human primary cells was approved by the IRB at the Case Western Reserve University Cancer Center.

### Compound Library Screen

For screening, HL-60 cells (5×10^5^ cells/ml) were cultured in 96-well plates. Cells were treated with 10 µM of compounds from the Prestwick collection (Prestwick). DMSO (0.1%) and ATRA (1 µM) were used as negative and positive controls respectively. After 5 days the differentiation of the cells was assessed by the nitroblue tetrazolium (NBT) reduction assay. To perform the NBT assay, 20 µl of a solution of NBT (5 mg/ml) and PMA 200 ng/ml (as a stimulant of the respiratory burst) was added to the HL-60 cells. The cells were incubated at 37°C for 30–40 minutes and the color of the wells was assessed. Those wells that displayed a visual color change from the production of blue insoluble formazan greater than the negative control where examined microscopically to determine the percentage of blue cells. At least 200 cells were counted for each positive well.

### Differentiation

NBT reduction was performed in a similar manner as described for the compound library screen. Immunophenotyping was performed by co-staining cells with CD11b-PE and CD14-FITC (Beckton Dickinson). The stained samples were run on a Beckman Coulter Cytomics FC 500 cytometer. For morphology assessment, cytospin preparations were made using a Shandon cytospin3 cytocentrifuge and the slides were stained with a modified wright-giemsa stain. For the combination drug studies, the Bliss synergy method was utilized to confirm the combination treatments led to synergistic differentiation.

### Cell proliferation and viability

Cell proliferation was assessed by manual counting at least 200 cells in 2 separate fields with a hematocytometer as well as with a Coulter counter. Cell viability was assessed by the trypan blue dye exclusion method.

### Clonogenic assays

Cells were treated with securinine or vehicle for 4 days. The cells were washed twice with PBS and 10000 viable THP-1, OCI-AML3 or HL-60 cells were dissolved in 3 ml of 0.30–0.35% Soft Agar (Noble Agar, Sigma) supplemented with 20% FBS. The cells were plated in gridded plates and incubated for 10 days in 37°C in a 5% CO2 incubator. After 10 days the colonies were counted under a light microscope. A minimum of 20 cells was required to be considered a colony. For the primary patient samples and normal human bone marrow cells, the cells were dissolved in methylcellulose supplemented with 20% FBS, SCF, G-CSF, GM-CSF and IL-3.

### Cell cycle analysis

Cells were treated for 24 hours and fixed at −20°C in 90% methanol. Cells were washed in PBS, treated with RNase A (final concentration 0.5 µg/ml) (Sigma) and stained with propidium iodide (50 µg/ml). The cells were kept at RT for 30–60 minutes and analyzed by flow cytometry.

### Gene Expression Array

HL-60 cells were treated with securinine (15 µM) for 24 hours, total RNA was isolated and cRNA was prepared using the Illumina Totalprep RNA amplification kit (Ambion). The cRNA was assessed with a Human-Ref-8 Illumina expression array and the data was analyzed using the Bead Studio Software (Illumina). Analysis was performed to identify genes that underwent at least a 2.5 fold change in expression and that had a known function in transcription using the Bead Studio Software.

### Real-Time PCR

Total RNA was isolated from cells treated with securinine or vehicle at indicated time points, using TRIzol reagent (Invitrogen). RNA was transcribed into cDNA using the Enhanced Avian RT First Strand Synthesis Kit (Sigma). Relative quantitative PCR was performed in triplicate using the FastStart SYBR Green Master (Roche Diagnostics) on an Applied Biosystems 7500 Fast Real-TimePCR System (see Figure S1 for primers and thermal conditions).

### Western blot analysis

Western blot analysis was performed with p21, p53, p-H2AX (Santa Cruz), p-Chk1 (Cell Signaling) and β-actin antibodies (Sigma). Cells were treated with securinine or vehicle for the indicated times and washed in PBS. Cells were centrifuged and lysed with a Triton containing lysis buffer. Protein lysates (50 µg per lane) were electrophoresed on SDS-polyacrylamide gels and then transferred to PVDF membranes (Millipore) using a wet transfer apparatus (Bio-Rad). The membranes were blocked, incubated with the indicated primary antibodies at 4°C overnight, and then the appropriate HRP conjugated secondary antibody. Protein bands were visualized by autoradiography after incubation with enhanced chemiluminescence reagent (Pierce).

### Lentiviral infection

Lentiviruses for shp21, shp53, shChk1 (Sigma) and the empty lentiviral vector, pLKO.1 (Open Biosystems) were packaged in 293T cells using packaging constructs pCMV-dR8.74 and pMD2G (Addgene). The shp21 and shp53 constructs were kindly provided by Dr. Mark Jackson. Supernatant media containing virus, collected at 48 h was concentrated 20 fold by precipitation with PEG overnight and supplemented with 4 µg/mL polybrene. OCI-AML3 cells were infected with virus containing media for 24 hours and then selected for stable infection with puromycin (1 µg/ml). Knockdown was confirmed by western blot (see Figure S3).

### Mouse Xenograft

6 week old female nude mice were injected bilaterally s.c. with 10×10^6^ HL-60 cells. Drug treatment was started 10 days after tumor cell injection. Palpable tumors were present for the established tumor model prior to initiating drug treatment. 15 mg/kg of securinine or vehicle (30 µL of DMSO and 70 µl of water ) were injected i.p. 2 or 3 times a day for 5 days followed by once a day for two days. This injection schedule was repeated for two additional weeks. The Case Western Reserve University Animal Research Committee approved all of the animal protocols used in this study.

## Supporting Information

Figure S1 **Real-time PCR primers and thermal conditions.**
(TIFF)

Figure S2 **Transcription-related genes exhibiting a 2.5 fold or greater change in expression after securinine treatment.**
(DOCX)

Figure S3 **OCI-AML3 cells exhibiting knockdown of Chk1, p53 and p21.** OCI-AML3 cells infected with a vector control or the indicated shRNA were lysed and the protein was analyzed by western blot. As the level of p21 protein expression is relatively low in the untreated OCI-AML3 cells, these cells were treated with doxorubicin to induce p21 expression.
(TIFF)

## References

[1] Estey E, Dohner H (2006). Acute myeloid leukaemia.. Lancet.

[2] American Cancer Society (2006). http://www.cancer.org/docroot/CRI/content/CRI_2_41x_What_Are_the_Key_Statistics_About_Acute_Myeloid_Leukemia_AML.asp?rnav=cri.

[3] Kell J (2004). Emerging treatments in acute myeloid leukaemia.. Expert Opin Emerg Drugs.

[4] Tallmann MS (2004). Curative therapeutic approaches to APL.. Ann Hematol.

[5] IaA A, Turova AD (1956). [Pharmacology of a new alkaloid securinine.].. Farmakol Toksikol.

[6] Duke J (1985).

[7] Beutler JA, Karbon EW, Brubaker AN, Malik R, Curtis DR (1985). Securinine alkaloids: a new class of GABA receptor antagonist.. Brain Res.

[8] Buravtseva GR (1958). [Result of application of securinine in acute poliomyelitis.].. Farmakol Toksikol.

[9] Copperman R, Copperman G, Der Marderosian A (1973). From Asia securinine–a central nervous stimulant is used in treatment of amytrophic lateral sclerosis.. Pa Med.

[10] Copperman R, Copperman G, Marderosian AD (1974). Letter: Securinine.. Jama.

[11] Dong NZ, Gu ZL, Chou WH, Kwok CY (1999). Securinine induced apoptosis in human leukemia HL-60 cells.. Zhongguo Yao Li Xue Bao.

[12] Rana S, Gupta K, Gomez J, Matsuyama S, Chakrabarti A (2010). Securinine induces p73-dependent apoptosis preferentially in p53-deficient colon cancer cells.. FASEB J.

[13] Lubick K, Radke M, Jutila M (2007). Securinine, a GABAA receptor antagonist, enhances macrophage clearance of phase II C. burnetii: comparison with TLR agonists.. J Leukoc Biol.

[14] Collins SJ, Ruscetti FW, Gallagher RE, Gallo RC (1979). Normal functional characteristics of cultured human promyelocytic leukemia cells (HL-60) after induction of differentiation by dimethylsulfoxide.. J Exp Med.

[15] Newburger PE, Chovaniec ME, Greenberger JS, Cohen HJ (1979). Functional changes in human leukemic cell line HL-60. A model for myeloid differentiation.. J Cell Biol.

[16] Collins SJ, Bodner A, Ting R, Gallo RC (1980). Induction of morphological and functional differentiation of human promyelocytic leukemia cells (HL-60) by componuds which induce differentiation of murine leukemia cells.. Int J Cancer.

[17] Guglielmo P, Pagano MC, Giustolisi R (1980). [The nitroblue tetrazolium activated test in the study of granulocytic function. Proposed methodology].. Boll Soc Ital Biol Sper.

[18] Newburger PE, Speier C, Borregaard N, Walsh CE, Whitin JC (1984). Development of the superoxide-generating system during differentiation of the HL-60 human promyelocytic leukemia cell line.. J Biol Chem.

[19] Wald DN, Vermaat HM, Zang S, Lavik A, Kang Z (2008). Identification of 6-benzylthioinosine as a myeloid leukemia differentiation-inducing compound.. Cancer Res.

[20] Alam S, Laughton DL, Walding A, Wolstenholme AJ (2006). Human peripheral blood mononuclear cells express GABAA receptor subunits.. Mol Immunol.

[21] Hill L, Holdsworth D, Small R (1976). Pharmacological investigations of virosecurinine.. P N G Med J.

[22] Pu H, Zhao J, Pong B, Xu S (2001). [Pharmacological comparison between virosecurinine and securinine].. Zhong Yao Cai.

[23] Rana S, Gupta K, Gomez J, Matsuyama S, Chakrabarti A (2010). Securinine induces p73-dependent apoptosis preferentially in p53-deficient colon cancer cells.. FASEB J.

[24] Meyer M, Rubsamen D, Slany R, Illmer T, Stabla K (2009). Oncogenic RAS enables DNA damage- and p53-dependent differentiation of acute myeloid leukemia cells in response to chemotherapy.. PLoS One.

[25] Asada M, Yamada T, Fukumuro K, Mizutani S (1998). p21Cip1/WAF1 is important for differentiation and survival of U937 cells.. Leukemia.

