# Supplementary figures and images for: Securinine, a Myeloid Differentiation Agent with Therapeutic Potential for AML
Source: PLoS One. 2011 Jun 24;6(6):e21203. doi: 10.1371/journal.pone.0021203 (PMC3123298; doi:10.1371/journal.pone.0021203)

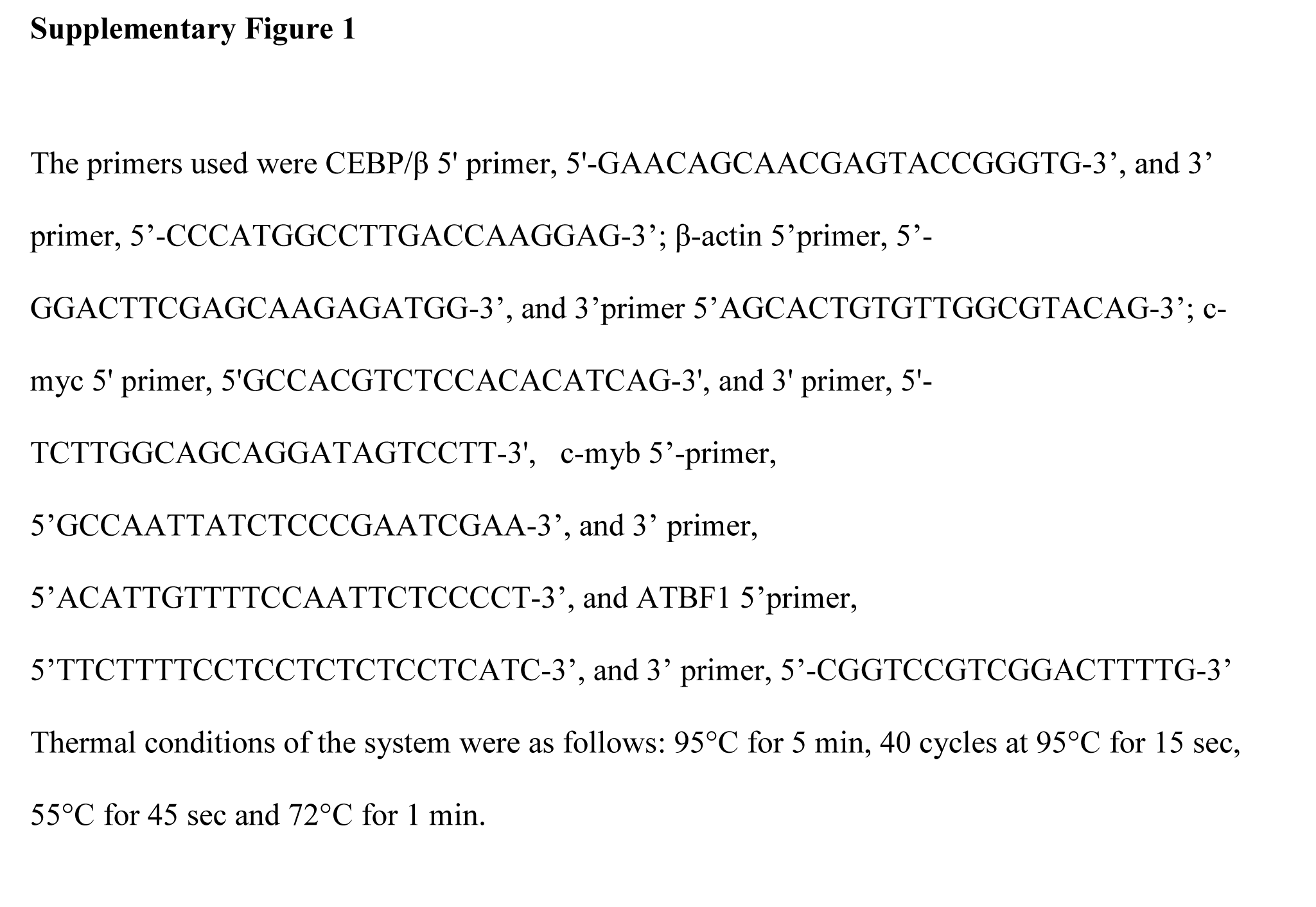

Supplement: Figure S1 — Real-time PCR primers and thermal conditions. (TIFF) [file pone.0021203.s001.tif]

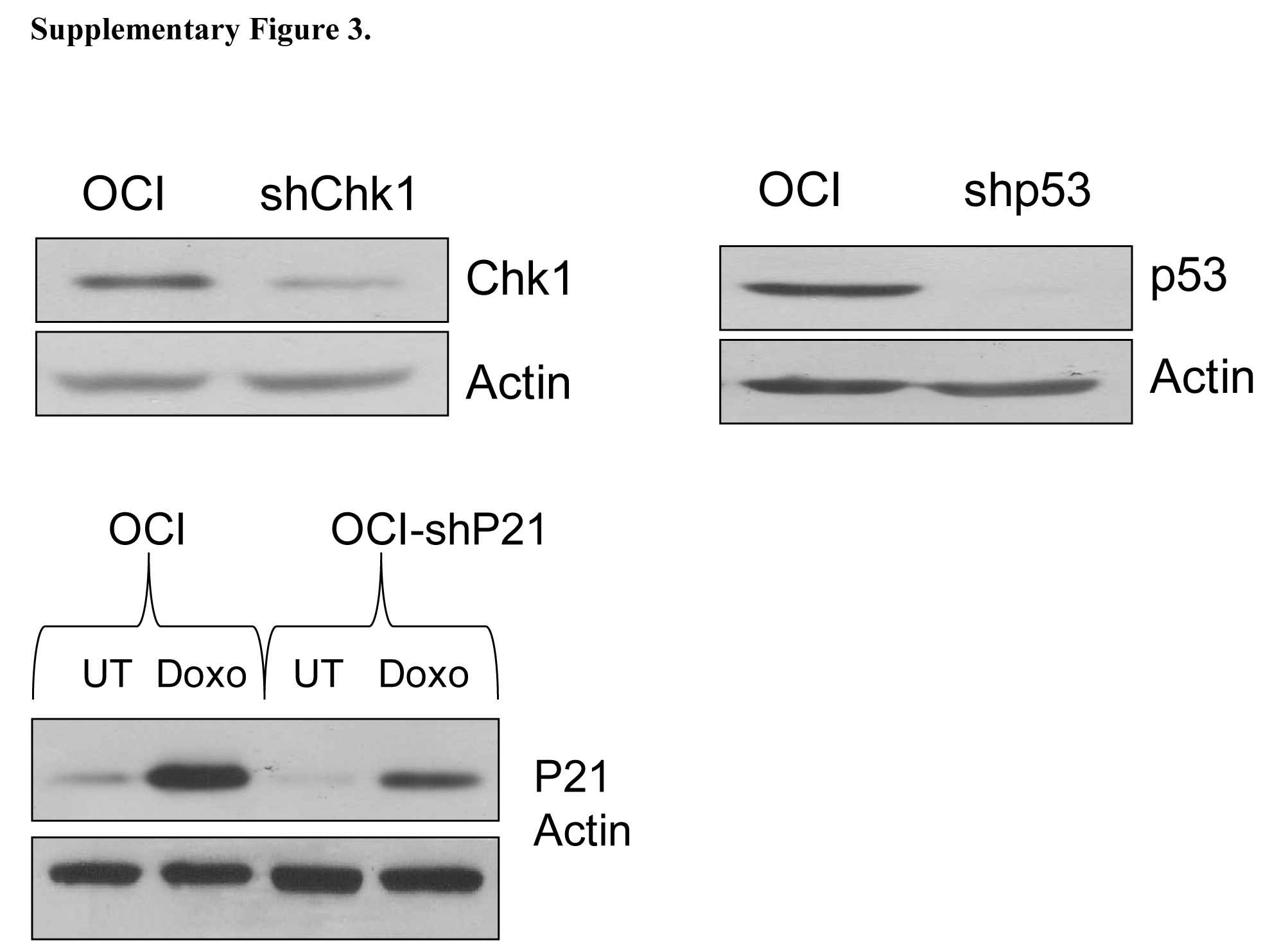

Supplement: Figure S3 — OCI-AML3 cells exhibiting knockdown of Chk1, p53 and p21. OCI-AML3 cells infected with a vector control or the indicated shRNA were lysed and the protein was analyzed by western blot. As the level of p21 protein expression is relatively low in the untreated OCI-AML3 cells, these cells were treated with doxorubicin to induce p21 expression. (TIFF) [file pone.0021203.s003.tif]
