# Supplementary material for: Securinine, a Myeloid Differentiation Agent with Therapeutic Potential for AML
Source: PLoS One. 2011 Jun 24;6(6):e21203. doi: 10.1371/journal.pone.0021203 (PMC3123298; doi:10.1371/journal.pone.0021203)
Supplement: Figure S2 — Transcription-related genes exhibiting a 2.5 fold or greater change in expression after securinine treatment. (DOCX) [file pone.0021203.s002.docx]

Supplementary Figure 2.

|  | | | | |
| --- | --- | --- | --- | --- |
|  |  |  |  |  |
| Upregulated genes | |  | Downregulated genes | |
| AFF4 | 3.65 |  | AFF3 | 0.40 |
| ARNTL | 2.59 |  | APEX1 | 0.19 |
| ATBF1 | 4.60 |  | BCL11A | 0.40 |
| BATF | 4.28 |  | BRCA2 | 0.10 |
| BAZ2B | 3.18 |  | CCRN4L | 0.33 |
| BCL3 | 15.53 |  | CITED4 | 0.36 |
| BCL6 | 17.12 |  | DACH1 | 0.39 |
| BHLHB2 | 2.72 |  | DEAF1 | 0.26 |
| BTBD4 | 5.98 |  | EAF2 | 0.01 |
| BTG2 | 30.00 |  | EBF3 | 0.38 |
| CARHSP1 | 3.31 |  | GATA2 | 0.33 |
| CASZ1 | 9.43 |  | HMGB1 | 0.36 |
| CD86 | 16.60 |  | IRF8 | 0.14 |
| CEBPB | 7.59 |  | ISL2 | 0.21 |
| CEBPD | 2.71 |  | KLF11 | 0.36 |
| CRK | 2.51 |  | KLP1 | 0.30 |
| CTNNB1 | 3.45 |  | MCM2 | 0.26 |
| DDIT3 | 4.74 |  | MCM3 | 0.01 |
| E2F2 | 2.64 |  | MCM6 | 0.26 |
| E2F7 | 3.58 |  | MYB | 0.37 |
| EGR1 | 21.04 |  | MYBBP1A | 0.40 |
| EGR2 | 10.46 |  | MYC | 0.19 |
| ELL2 | 2.62 |  | NFIA | 0.39 |
| ENPP2 | 2.54 |  | NFKBIL2 | 0.23 |
| ETS1 | 3.00 |  | NSBP1 | 0.20 |
| ETS2 | 2.53 |  | PEG3 | 0.37 |
| ETV5 | 3.14 |  | PMS1 | 0.38 |
| FLJ37970 | 2.96 |  | POLR1B | 0.19 |
| FMNL2 | 3.78 |  | POLR2K | 0.10 |
| FOS | 2.72 |  | POLR3B | 0.12 |
| GAS7 | 9.81 |  | PRAF1 | 0.32 |
| HBP1 | 4.07 |  | PTMA | 0.10 |
| HIF1A | 2.52 |  | RUVBL1 | 0.25 |
| HIPK2 | 6.07 |  | RUVBL2 | 0.15 |
| HIVEP1 | 4.33 |  | SIX4 | 0.40 |
| ID2 | 7.96 |  | SMARCA3 | 0.18 |
| IFI16 | 8.92 |  | SOX12 | 0.39 |
| IRF1 | 2.50 |  | TCEA3 | 0.40 |
| IRF7 | 12.29 |  | TCEAL1 | 0.26 |
| ISGF3G | 3.06 |  | TFAP4 | 0.28 |
| JUN | 2.94 |  | TGIF2 | 0.02 |
| KLF10 | 2.55 |  | TRIP13 | 0.14 |
| KLF6 | 3.03 |  | TWISTNB | 0.15 |
| LITAF | 4.23 |  | WT1 | 0.38 |
| LOC153222 | 4.04 |  | ZFP1 | 0.34 |
| MAFB | 84.57 |  | ZHX3 | 0.15 |
| MLL3 | 2.66 |  | ZNF323 | 0.34 |
| MLLT7 | 3.09 |  | ZNF343 | 0.16 |
| MSC | 4.90 |  | ZNF485 | 0.32 |
| MSRB2 | 3.76 |  | ZNF519 | 0.25 |
| MTF1 | 2.93 |  | ZNF589 | 0.33 |
| MXD1 | 8.03 |  | ZNF658 | 0.27 |
| NAB1 | 2.57 |  | ZNF714 | 0.22 |
| NCOA1 | 3.15 |  |  |  |
| NCOA3 | 2.74 |  |  |  |
| NFATC1 | 4.31 |  |  |  |
| NFIL3 | 2.73 |  |  |  |
| NFKB2 | 8.88 |  |  |  |
| NFKBIE | 4.28 |  |  |  |
| NOTCH1 | 8.73 |  |  |  |
| OLIG1 | 3.29 |  |  |  |
| PBX3 | 3.48 |  |  |  |
| PCAF | 8.60 |  |  |  |
| PCGF2 | 4.51 |  |  |  |
| PHTF1 | 3.59 |  |  |  |
| PIR | 6.59 |  |  |  |
| POU2F2 | 3.42 |  |  |  |
| PPARD | 7.88 |  |  |  |
| PPARG | 5.60 |  |  |  |
| PRDM1 | 7.55 |  |  |  |
| PRIC285 | 96.10 |  |  |  |
| RELB | 11.43 |  |  |  |
| RFX2 | 2.67 |  |  |  |
| RKHD3 | 16.34 |  |  |  |
| RRAGC | 2.67 |  |  |  |
| RUNX3 | 9.48 |  |  |  |
| RXRA | 2.81 |  |  |  |
| RYBP | 3.05 |  |  |  |
| SERTAD1 | 6.95 |  |  |  |
| SKIL | 11.76 |  |  |  |
| SMAD7 | 4.71 |  |  |  |
| SOD2 | 3.05 |  |  |  |
| SOX13 | 13.48 |  |  |  |
| SP100 | 3.11 |  |  |  |
| SP140 | 10.53 |  |  |  |
| SQSTM1 | 37.55 |  |  |  |
| STAT3 | 5.01 |  |  |  |
| STAT5A | 3.63 |  |  |  |
| TAF13 | 6.36 |  |  |  |
| TFAP2A | 3.99 |  |  |  |
| TFE3 | 5.13 |  |  |  |
| TLE3 | 8.72 |  |  |  |
| TRPS1 | 22.98 |  |  |  |
| TSC22D2 | 2.69 |  |  |  |
| TSC22D3 | 2.66 |  |  |  |
| VDR | 2.74 |  |  |  |
| ZBTB7B | 2.64 |  |  |  |
| ZFP36L1 | 76.95 |  |  |  |
| ZNF20 | 8.85 |  |  |  |
| ZNF217 | 3.16 |  |  |  |
| ZNF230 | 3.15 |  |  |  |
| ZNF319 | 3.28 |  |  |  |
| ZNF447 | 3.89 |  |  |  |
| ZNF641 | 4.82 |  |  |  |
| ZNF651 | 2.61 |  |  |  |
